# Poly (ADP-ribose) polymerase inhibitor, an effective radiosensitizer in lung and pancreatic cancers

**DOI:** 10.18632/oncotarget.15464

**Published:** 2017-02-17

**Authors:** Kedar Hastak, Steven Bhutra, Renate Parry, James M. Ford

**Affiliations:** ^1^ Department of Medicine, Division of Oncology, Stanford University, Stanford CA 94305, USA; ^2^ Department of Biology, Stanford University, Stanford CA 94305, USA; ^3^ Current address: Department of Medicine, Advocate Illinois Masonic Medical Center, Chicago, IL 60657, USA; ^4^ Varian Medical Systems, Palo Alto, CA 94306, USA

**Keywords:** PARP inhibitor, fractionated radiation, combination therapy, radiosensitizer, lung cancer

## Abstract

The development of stereotactic body radiation therapy (SBRT) has revolutionized radiation therapy for lung cancers and is an emerging treatment option for pancreatic cancers. However, there are many questions on how to optimize its use in chemoradiotherapy. The most relevant addition to radiotherapy regimens are inhibitors of DNA repair and DNA damage response pathways. One such class of agents are inhibitors of poly (ADP-ribose) polymerase (PARP). In this study we examined the effects of the PARP inhibitor LT626 in combination with ionizing radiation in lung and pancreatic cancers. Our study demonstrated that combination treatment with LT626 and radiation effectively inhibited growth in lung and pancreatic cancer cell lines, better than individual treatment alone. Combination treatment also increased expression of γH2AX and 53BP1 foci and upregulated expression of phosphorylated ATM, ATR and their respective kinases. Using *in vivo* lung cancer xenograft models we demonstrated that LT626 functioned as an effective radiosensitizer during fractionated radiation treatment, leading to significant decrease in tumor burden and doubling the median survival compared to control group. Overall our *in vitro* and *in vivo* studies showed that PARP inhibitor LT626 acted synergistically with radiation in lung and pancreatic cancers.

## INTRODUCTION

Lung cancer remains the leading cause of cancer incidence and mortality worldwide [1]. According to the American Cancer Society in 2016, an estimated 158,080 Americans are expected to die from lung cancer [2]. Non-small cell lung cancer (NSCLC) accounts for 80–90% of all lung cancers and the standard of care for early-stage NSCLC is surgery. However, surgery is not a feasible option for most NSCLC patients who either cannot tolerate surgical stress or postoperative recovery, or for whom the disease burden is too extensive. Furthermore, clinical trials in NSCLC patients found that treatment based on a broad use of cytotoxic chemotherapies, with third generation platinum based drugs had reached its therapeutic plateau [3]. Studies have shown that for locally advanced NSCLC, conventional fractionated radiotherapy with concurrent chemotherapy was better than simple radiotherapy or sequential radiochemotherapy [4, 5].

Pancreatic cancer now ranks below only lung cancer and colorectal cancer in the number of cancer-related deaths annually. In 2016, there will be an estimated 53,070 new diagnosis and 41,780 deaths from pancreatic cancer in the United States [2]. Pancreatic cancer mortality rates have not substantially declined and the incidence is increasing in low and middle resource countries. Similar to lung cancer, more than 80% of cancers are detected at advanced stages where they cannot be removed surgically and are thus incurable [6]. Even management of locally advanced pancreas cancer is challenging and many times surgery cannot be performed due to its morbidity and improbability of cure. Patient thus may undergo intensive treatment with radiation, chemotherapy or both.

Many studies have demonstrated that stereotactic body radiation therapy (SBRT) can be an effective treatment modality for early-stage lung cancers [7–9]. Despite promising clinical results and an enthusiasm for expanding use of SBRT to other organs sites [10] the biological mechanisms involved in SBRT are still poorly understood. Therefore, there is a need to identify effective radiation sensitizers and to understand the mechanism and treatment response by using *in vitro* and *in vivo* models in fractionated radiation settings. This is an ideal setting for involving targeted agents which can induce DNA damage with radiation treatment.

Poly (ADP-ribose) polymerase 1 (PARP1) and PARP2 are important DNA damage sensors. PARP1 binds damaged DNA via its N-terminal zinc motifs, which activates its catalytic C-terminal domain to hydrolyze NAD and produce linear and branched PAR chains that can extend over hundreds of ADP-ribose units [11–13]. PARP1 and PARP2 bind rapidly at the site of DNA damage and help in the resealing of single stranded DNA breaks during break excision repair and for the repair of topoisomerase 1 cleavage complex [14–16].

PARP inhibitors first entered clinical trials in 2003 in combination with the mono-methylating agent temozolomide in patients with advanced solid tumors [17]. Subsequent pre-clinical studies in BRCA1 deficient cells (defective in homologous recombination) demonstrated the concept of synthetic lethality [18–20]; later other studies showed that PARP inhibitors are also effective in cells with “BRCAness” phenotype, which is defined as traits that sporadic cancers share with those occurring in BRCA1 mutation carriers, often due to defects in DNA repair. Several cancers like triple negative breast cancers and ovarian cancers with wild type BRCA1 also exhibit sensitivity to PARP inhibitors [21–25].

In the present study we sought to evaluate if a PARP inhibitor acts synergistically with radiation treatment in lung and pancreatic cancers. We also wanted to assess the use of PARP inhibitors as sensitizers in fractioned radiation treatment. To do so, we performed *in vitro* studies using two lung and two pancreatic cancer cell lines. We showed that these cell lines are sensitive to PARP inhibition and radiation and to the combination treatment of a PARP inhibitor and radiation. We also used *in vivo* lung cancer xenograft models to demonstrate that PARP inhibitors can be used as sensitizers in fractionated radiation treatment.

## RESULTS

### Sensitivity of lung and pancreatic cell lines to LT626 and radiation

**Figure 1:**
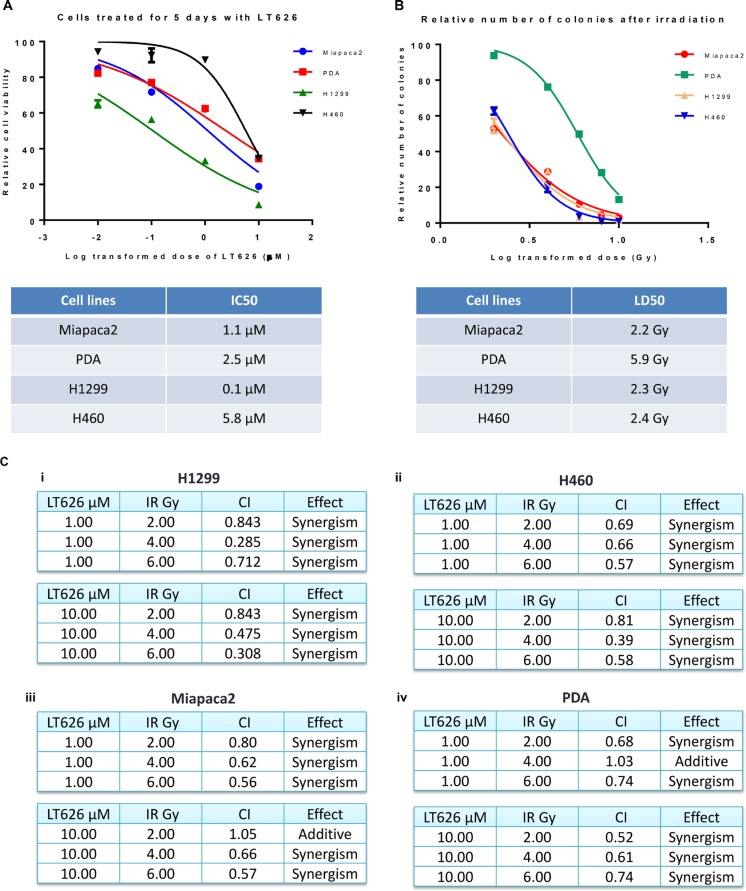
Sensitivity to LT626 or radiation in lung and pancreatic cancer cell lines Pancreatic (Miapaca2, PDA) and lung (H1299, H460) cancer cell lines were treated with 0–10 μM of LT626 or 1–10 Gy of radiation. (**A**) Cell viability was measured after 5 days of LT626 treatment by MTT assay and IC_50_ was calculated using Nonlinear regression (curve fit) model by PRISM. (**B**) After radiation cells were plated for colony formation assay and IC_50_ was calculated. (**C**) Combination index values for cell lines treated in combination with LT626 and radiation. A non-constant drug ratio model was used when treating the cells, wherein concentration of LT626 was kept constant and the amount of radiation was varied. After treatment combination index (CI) was calculated using Calcusyn software (from Biosoft). (i) CI values for H1299 cell line treated with 1 μM LT626 and 2–6 Gy radiation (top table) or 10 μM LT626 and 2-6 Gy radiation. (ii) H460 cell line, (iii) Miapaca2 cell line and (iv) PDA cell line. Experiments were done three independent times in triplicate. Graph represents cell viability in log scale ± SD.

Previous studies from our laboratory showed that PARP inhibitor effectively targeted triple negative breast cancer cells irrespective of their BRCA1 status [23]. Furthermore, our laboratory also demonstrated that PARP inhibitor LT626 synergizes with cisplatin, oxaliplatin, and SN-38 in colorectal cancer cell lines [26]. Therefore we wanted to study if a PARP inhibitor can be used to target lung and pancreatic cancers. We treated two pancreatic cancer cell lines (Miapaca2, PDA) and lung cancer cell lines (H1299, H460) with the PARP inhibitor, LT626 (0–10 μM) for five days and cell viability was measured by MTT assay. All four cell lines were sensitive to LT626 as shown in Figure 1A. Lung cancer cell line H1299, which is p53 null, was slightly more sensitive than lung cancer cell line H460 which expresses wild type p53. Mouse derived pancreatic cell line PDA exhibited a slightly higher IC_50_ than human derived Miapaca 2 (Figure 1A table). Next we tested all four cell lines for radiation sensitivity. Cells were irradiated with 0–10 Gy and sensitivity tested by colony formation assay. As shown in Figure 1B Miapaca2, H1299 and H460 were much more sensitive to radiation than PDA.

### Synergism between LT626 and radiation

Our results show that lung and pancreatic cell lines are sensitive to LT626 and radiation when used individually. However, to determine if LT626 can act as a radiosensitizer we treated Miapaca2, PDA, H1299 and H460 cell lines with a combination of LT626 and radiation followed by colony formation assay. For this study we used a non-constant ratio model wherein cells were either treated with 1 or 10 μM of LT626 followed by 2–6 Gy of radiation. CalcuSyn software was used to calculate combination index (CI) and plot normalized isobolograms. CI < 1, CI = 1 and CI > 1 quantitatively indicate synergism, additivity and antagonism respectively. We also calculated the linear coefficient r value to estimate the accuracy of measurement, and all our experiments had an r value > 0.90 for median effect plot.

As shown in Figure 1C (i and ii) lung cancer lines H1299 and H460 exhibited synergism for all different combinations of LT626 and radiation. Even pancreatic cell lines showed synergism with most of the combinations (Figure 1C iii and iv) with an additive effect seen in one combination treatment in PDA cells. There was no antagonism observed in any of the combination doses used. These results demonstrate that LT626 is a potent radiosensitizer and can be used in combination with radiation.

### Fractionated radiation scheme in treating lung and pancreatic cell lines

**Figure 2:**
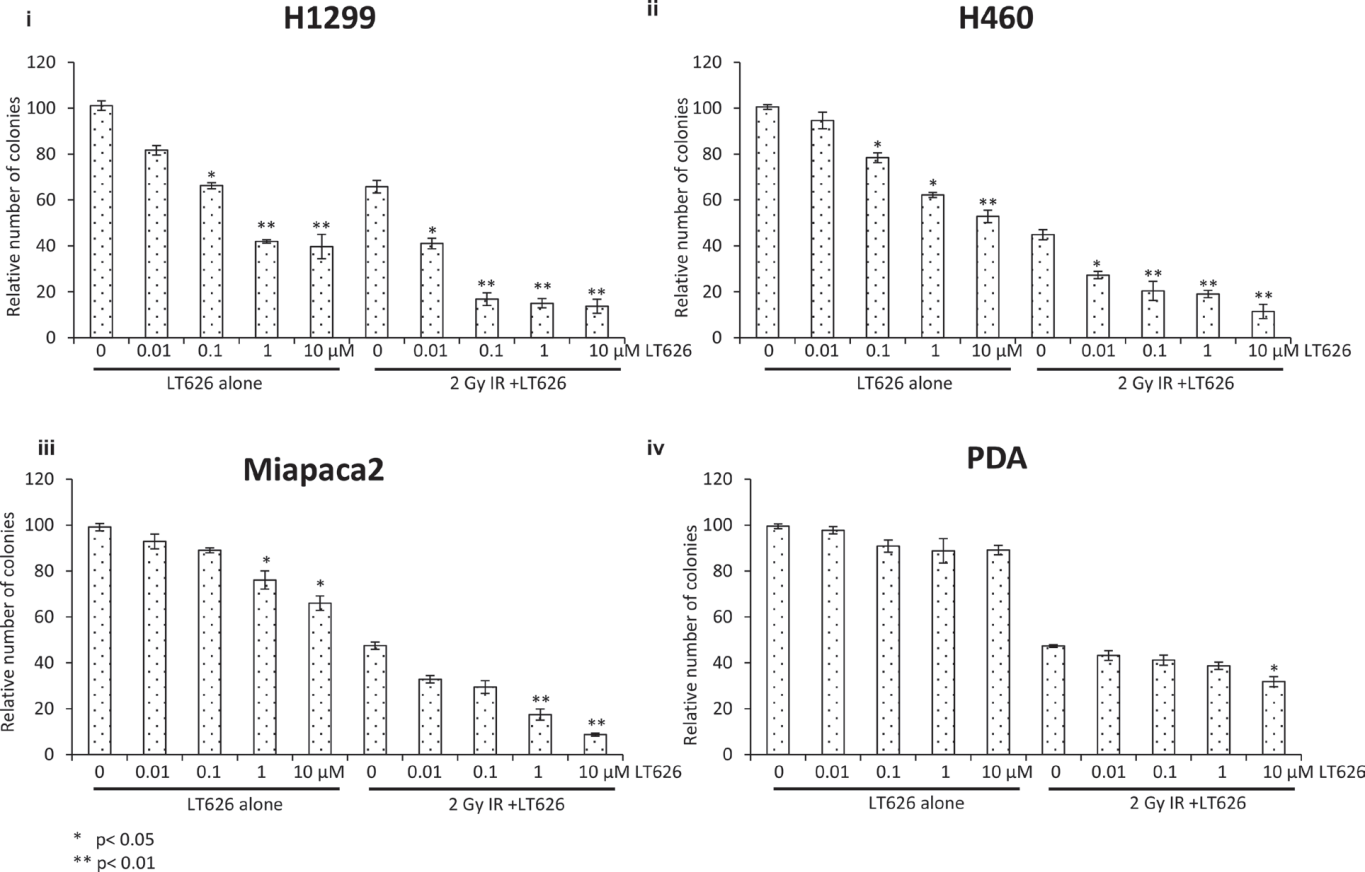
Fractionated radiation scheme Pancreatic and lung cancer cell lines were treated with either LT626 alone (0.01–10 μM) or 2 Gy of radiation alone or combination of LT626 and radiation. For combination treatment cells were pretreated with LT626 for 30 min followed by radiation. Cells were treated for 3 consecutive days with this treatment regimen, following treatment cells were plated for colony formation assay as described. Relative number of colonies standardized to control was plotted for each cell line. Experiments were done three independent times in triplicate. (i) H1299 cell line (ii) H460 cell line (iii) Miapaca2 cell line and (iv) PDA cell line. Bars represent cell viability ± SD. Statistical analysis to demonstrate significance was performed by using Student's *T*-test.

Radiation as a treatment modality in humans is usually given over a period of many days rather than in just one dose. To mimic the effect of fractionated radiation we treated lung and pancreatic cancer cell lines with combination of LT626 and radiation for three consecutive days. Cells were either treated with LT626 (0–10 μM) or 2 Gy radiation alone. For combination treatment, cells were pretreated for 30 minutes with LT626 followed by irradiation. After the last day of treatment cells were plated for colony formation assay. LT626 pretreatment conditions were optimized based on standardization experiments (data not shown) and 30 minutes pretreatment with LT626 was found to be ideal. As shown in Figure 2 pancreatic cell lines Miapaca2 and PDA (iii and iv respectively) were not very sensitive to LT626 treatment alone. Two Gy of radiation alone did show inhibition in cell growth, however pretreatment with LT626 followed by irradiation further inhibited cell growth which was significant (*p* < 0.05 and *p* < 0.01). As for PDA cells (iv) pretreatment with 10 μM LT626 showed some combinatorial effect, but lower concentrations of LT626 were not that effective. Lung cancer cells H1299 and H460 also showed similar results (i and ii respectively) wherein pretreatment of cells with 0.01 μM of LT626 was sufficient to enhance the effect of radiation.

### DNA damage in lung and pancreatic cell lines after combination treatment

**Figure 3:**
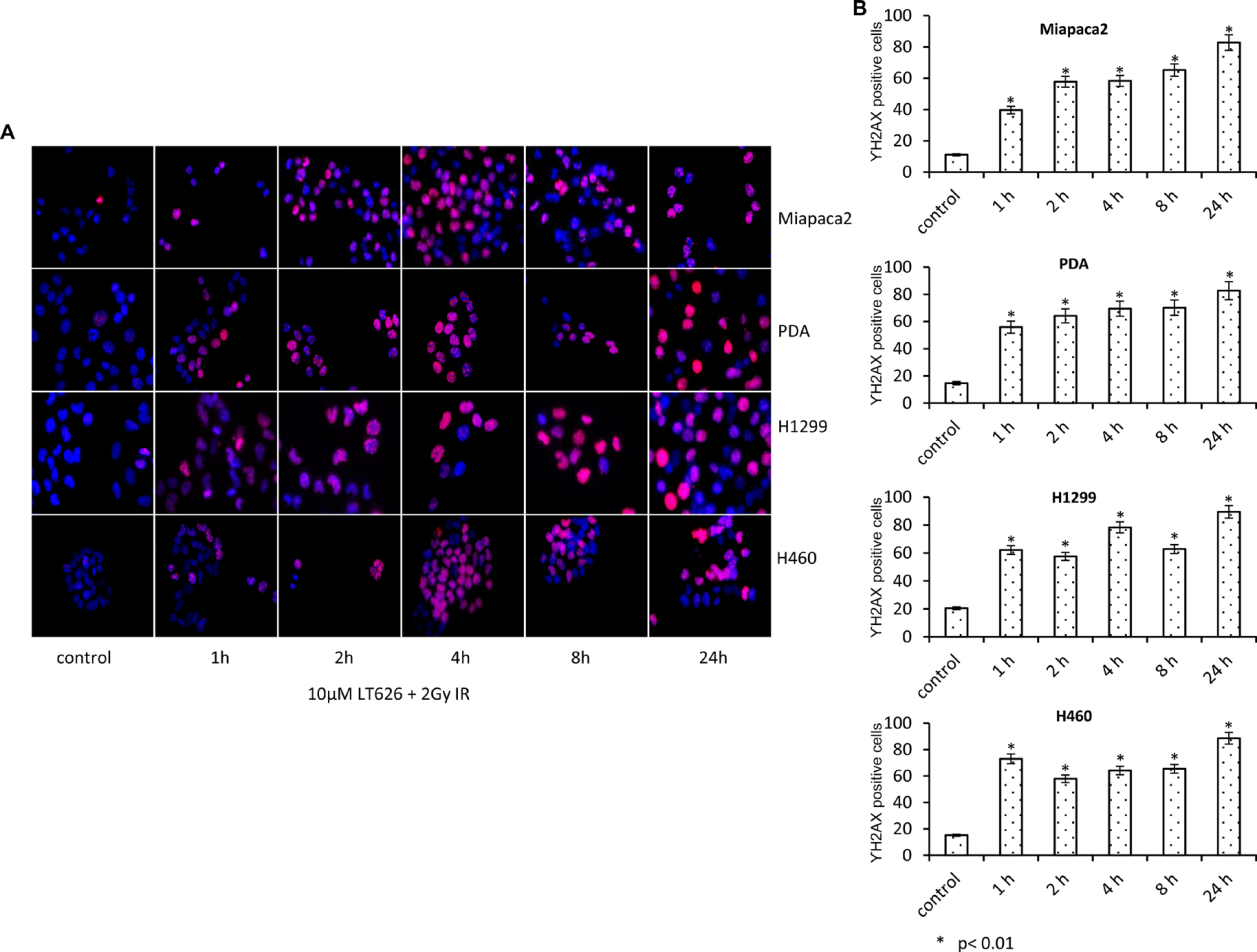
Increased expression of γH2AX after combination treatment Pancreatic and lung cancer cell lines were treated with a combination of 10 μM LT626 and 2 Gy irradiation. Following treatment cells were fixed and stained for γH2AX. (**A**) Representative staining of γH2AX positive cells, with punctate nuclear staining. (**B**) At least 100 cells were counted and only cells with more than 3 punctate γH2AX foci were considered positive. γH2AX positive cells relative to control were plotted. Experiments were done three independent times in triplicate. Bars represent mean ± SD. Statistical analysis to demonstrate significance was performed by using Student's *T*-test.

Since PARP plays an important role in DNA repair while radiation causes significant DNA damage we investigated the effect of LT626 and radiation on DNA damage by staining for γH2AX foci which accumulate at the site of broken DNA. To study γH2AX foci, cells were treated with 10 μM LT626 or 2 Gy of radiation or combination of 10 μM LT626 and 2 Gy of radiation for 1–24 h. Our studies showed that individual treatments led to increased γH2AX nuclear foci formation (Supplementary Figure 1), wherein maximum γH2AX foci after LT626 treatment were observed after 24 h of treatment while in radiation treated group γH2AX foci formation peaked by 2–4 h and then dropped by 24 h. However, in the combination treated group (Figure 3A) γH2AX foci accumulated as function of time. The number of γH2AX foci positive cells were counted for each cell line (Figure 3B) and there was more than a 2-fold increase after treatment which was statically significant, *p* < 0.01.

**Figure 4:**
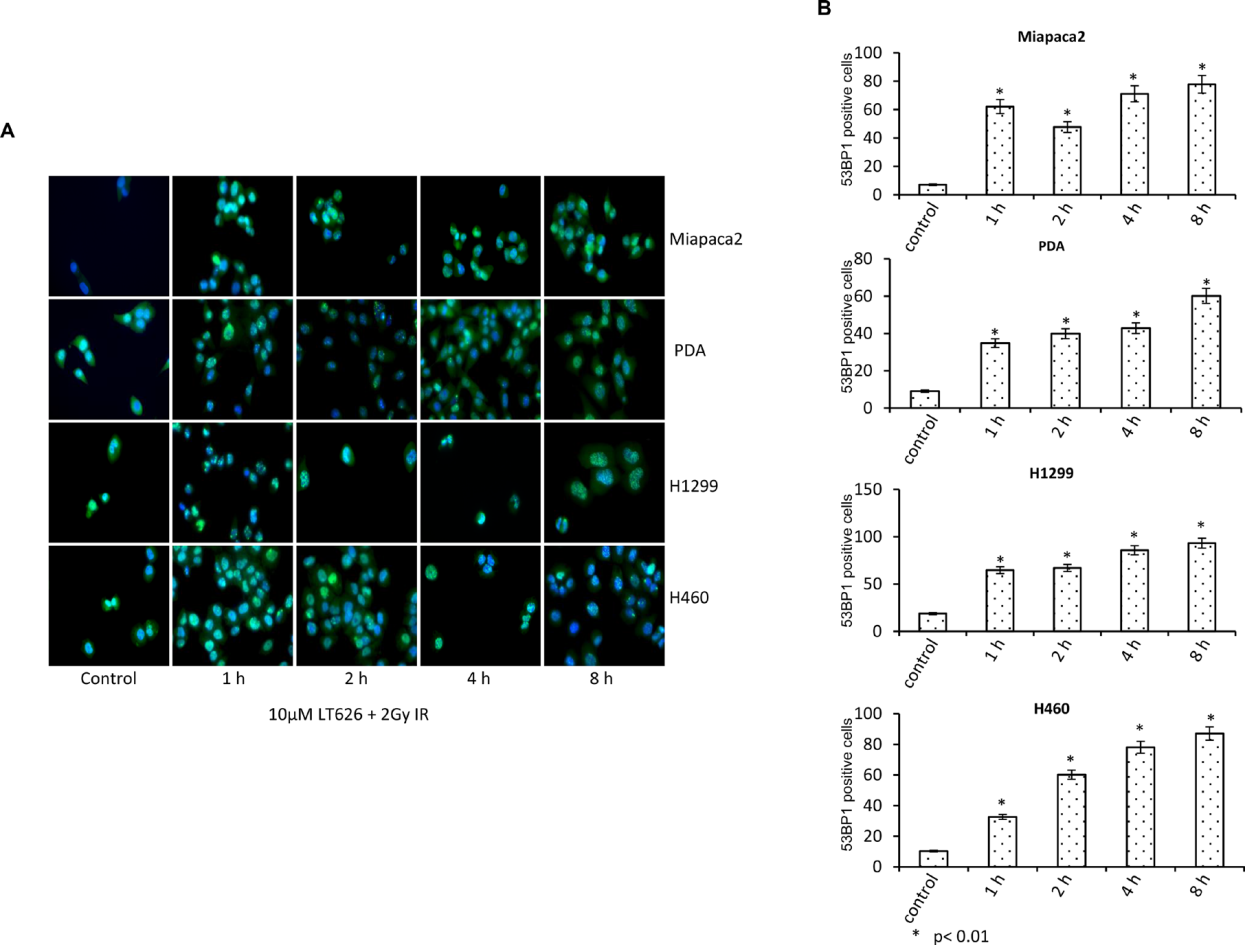
Expression of 53BP1 after combination treatment Pancreatic and lung cancer cell lines were treated with a combination of 10 μM LT626 and 2 Gy irradiation. Following treatment cells were fixed and stained for 53BP1. (**A**) Representative staining of 53BP1 positive cells, with punctate nuclear staining. (**B**) At least 100 cells were counted and only cells positive for punctate nuclear 53BP1 staining were considered positive. 53BP1 positive cells relative to control were plotted. Experiments were done three independent times in triplicate. Bars represent mean ± SD. Statistical analysis to demonstrate significance was performed by using Student's *T*-test.

53BP1 is a DNA damage checkpoint protein, with a key role in DNA repair response and checkpoint control. Upon formation of DNA double stranded breaks, 53BP1 rapidly redistributes from a diffuse nuclear localization to discrete foci that co-localize with phosphorylated histone H2AX and other repair proteins including BRCA1. We therefore investigated the expression of 53BP1 after combination treatment with LT626 and radiation. As shown in Figure 4A, combination treatment increased the expression of nuclear 53BP1 foci as function of time in both pancreatic and lung cancer cell lines. Individual treatment also increased 53BP1 foci with time; however there was no significant difference in the number of foci positive cells among individual treatments (Supplementary Figure 2) and combination treatment further enhanced the number of foci than LT626 or radiation alone. 53BP1 positive cells were counted for each cell line (Figure 4B) and similar to γH2AX there was 2-fold increase after treatment (*p* < 0.01, student *T*-test).

### Combination treatment increased survival in lung cancer xenograft models

**Figure 5:**
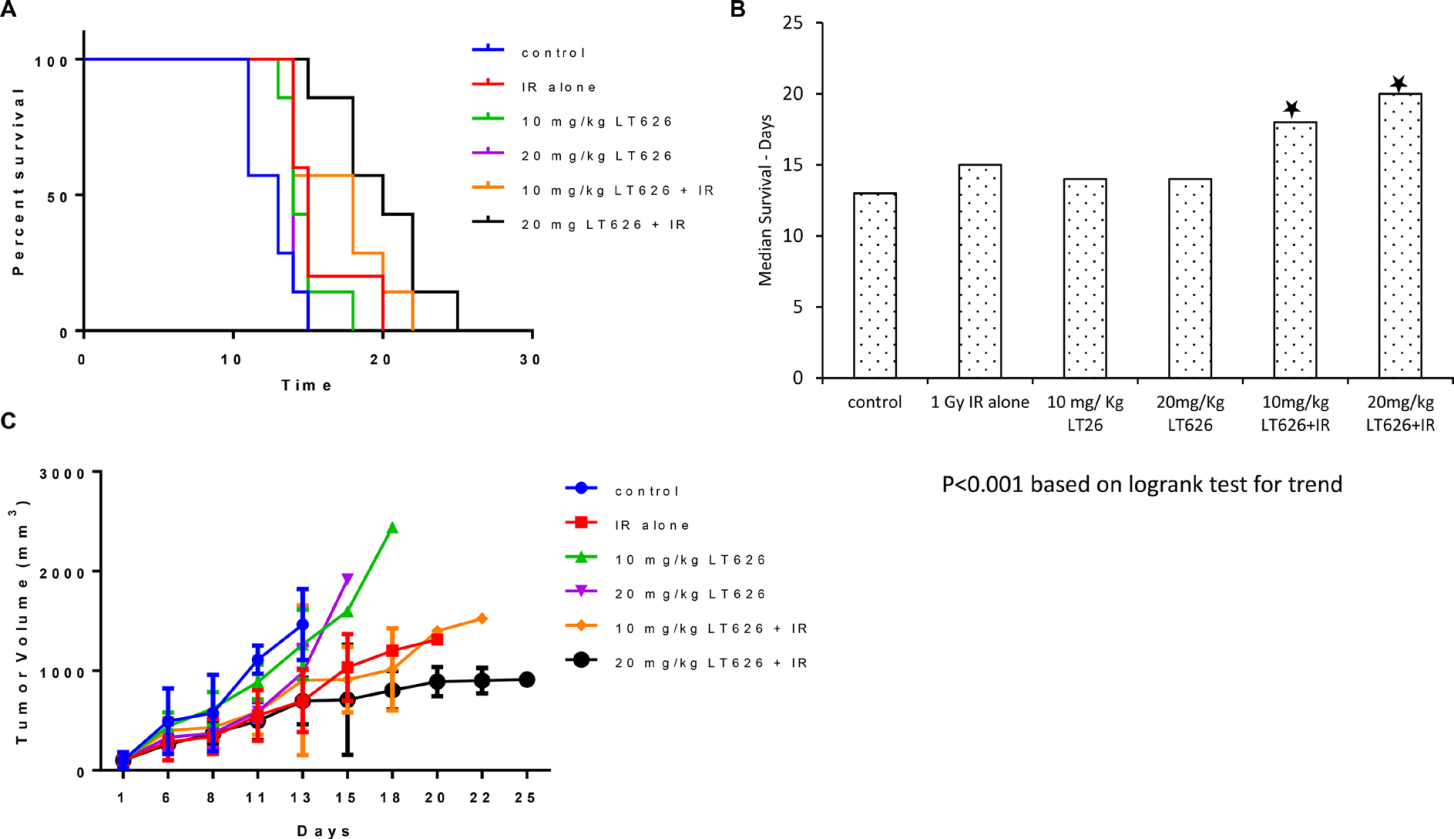
Overall survival and tumor volume after combination treatment in H460 xenograft model Mice were divided into 6 groups–control, 1 Gy IR alone, 10 mg/kg LT626, 20 mg/kg LT626, IR+10 mg/kg LT626 and IR+20 mg/kg LT626. Following 5 days of treatment (**A**) Kaplan-Meier survival curve for mice treated with LT626, radiation or combination of LT626 and radiation. (**B**) Mean survival based on the values from Kaplan-Meier curve. (**C**) Tumor volume standardized to control were plotted using PRISM, day zero is tumor volume measured one day before the 5 consecutive day treatment was started. Graph represents tumor volumes ± SD. Statistical analysis using Logrank test for trend was performed to demonstrate significance (*P* < 0.001) between control and treated groups.

Our *in vitro* data showed that LT626 and radiation is synergistic in inhibiting cell growth in lung and pancreatic cancers. We therefore wanted to study if there is similar synergism between LT626 and radiation in mouse tumor models. For this study we used two lung cancer cell lines (H1299 and H460). Female nude mice bearing H1299 or H460 xenograft tumors were treated for 5 consecutive days with LT626 alone (p.o) or radiation or combination of LT626 and radiation. For combination treatment, mice were administered LT626 30 minutes prior to radiation. As shown in Figure 5A and 5B, for H460 xenograft model individual treatment only slightly increased the overall survival, but mice treated with combination of 10 mg/kg LT626 + radiation and 20 mg/kg LT626 + radiation had significant increase in overall survival. The median survival increased from 13 days for control group to 18 days and 20 days for 10 mg/kg LT626 + IR and 20 mg/kg LT626 +IR group respectively (*P* < 0.01). Furthermore, overall tumor burden was significantly lower in combination treatment groups compared to control (*p* < 0.01) (Figure 5C).

**Figure 6:**
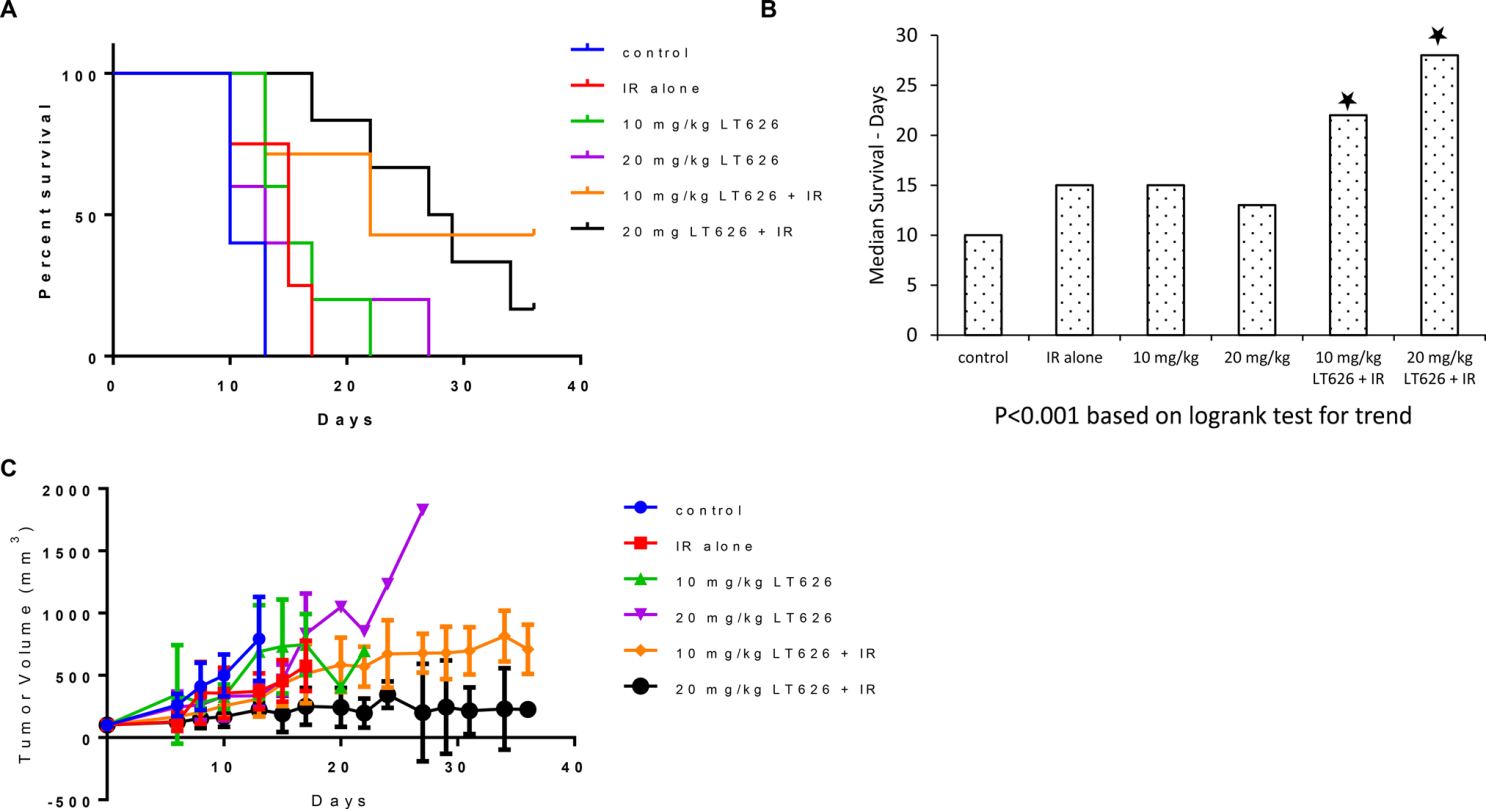
Overall survival and tumor volume after combination treatment in H1299 xenograft model Mice were divided into 6 groups–control, 1 Gy IR alone, 10 mg/kg LT626, 20 mg/kg LT626, IR+10 mg/kg LT626 and IR+20 mg/kg LT626. Following 5 days of treatment: (**A**) Kaplan-Meier survival curve for mice treated with LT626, radiation or combination of LT626 and radiation. (**B**) Mean survival based on the values from Kaplan-Meier curve. (**C**) Tumor volume standardized to control were plotted using PRISM, day zero is tumor volume measured one day before the 5 consecutive day treatment was started. Graph represents tumor volumes ± SD. Statistical analysis using Logrank test for trend was performed to demonstrate significance (*P* < 0.001) between control and treated groups.

Combination treatment had a much more pronounced effect in the H1299 xenograft model, wherein 10 mg/kg LT626 + IR and 20 mg/kg LT626 +IR groups had mice alive at the end of the study (Figure 6A). The medial survival more than doubled to 22 and 28 days for the two combination groups compared to 10 days for the control groups (Figure 6B). The overall tumor burden was also significantly lower in combination treatment groups compared to control. The 20 mg/kg LT626 + IR group had the lowest tumor burden (Figure 6C).

### Differential expression of ATR and ATM in lung cancer cells after LT626 and radiation treatment

**Figure 7:**
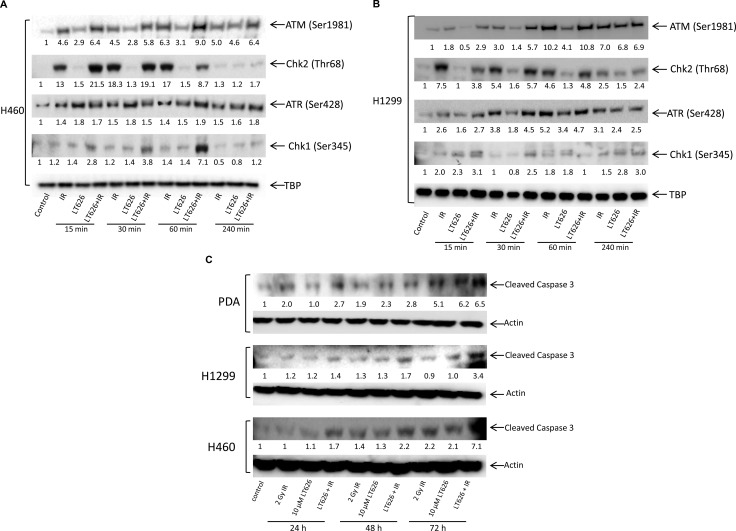
Expression of ATM, ATR, Chk1, Chk2 and cleaved caspase 3 after treatment Cells were treated with either 10 μM LT626, 2 Gy IR or with combination of LT626 and IR for desired time. (**A**) Expression of phosphorylated ATM, Chk2, ATR and Chk1 in H460 cell line. (**B**) Expression of phosphorylated ATM, Chk2, ATR and Chk1 in H1299 cell line. TBP was used as a nuclear loading control (**C**) Expression of cleaved caspase 3 following treatment in H460, H1299 and PDA cell lines. Quantification of blots was carried out using ImageJ software and compared to control; values are shown below respective blots.

ATM and ATR are two important DNA damage checkpoint kinases that delay or arrest cell cycle progression in response to DNA damage. Studies have shown that phosphorylation of H2AX is dependent on ATM and ATR. We therefore investigated the expression of phosphorylated ATM and ATR and their respective kinases Chk2 and Chk1 in nuclear extracts of H1299 and H460 cell lines treated with LT626, radiation or combination treatment for 15–240 minutes. As shown in Figure 7, radiation and combination treatment led to phosphorylation of ATM (Ser1981) and Chk2 (Thr68) within 15 minutes of treatment in both H460 cell line (7A) and H1299 cell line (7B). LT626 (alone) treatment did not substantially phosphorylate ATM or Ckh2. On the other hand, LT626 treatment led to increased phosphorylation of ATR (Ser428) and Chk1 (Ser345). Interestingly combination treatment increased the expression of phosphorylated ATR and ATM in both cell lines (Figure 7A and 7B). Figure 7C shows that combination treatment increased the expression of cleaved caspase 3 by 24 h, whereas single treatment modalities increased cleaved caspase 3 expression only by 48 and 72 h after treatment in pancreatic and lung cancer cells.

## DISCUSSION

Treatment failure of the primary lesion of NSCLC has negative effects on progression free survival, metastasis free survival and overall survival [27]. Increasing the tumor radiation dose could increase the local control [28, 29]. However, only increasing the radiotherapy dose alone is not sufficient to increase overall survival as shown in the study RTOG0617 [30]. Thus other important factors like radiosensitizers/ combination therapy might obtain more benefits.

Molecules targeting DNA repair pathways have shown great potential to sensitize tumor cells to both chemotherapy and radiotherapy by increasing their cytotoxicity [31]. This makes PARP inhibitors potential candidates as radiosensitizers. The radiosensitization effects of PARP inhibitors have been shown to be specific to cells in the S-phase of the cell cycle [32] and are due to the collision of the persisting single strand breaks with replication forks and the formation of a lethal DNA double strand break [33, 34]. Therefore, there is a strong rational for the use of PARP inhibition in association with SBRT for treatment of lung and pancreatic cancers.

In our current study we show that lung and pancreatic cancer cell lines are sensitive to the PARP inhibitor LT626 and irradiation. Since PARP plays an important role in response to DNA damage and radiation leads to double stranded breaks we wished to examine whether LT626 acts synergistically with radiation. To study the combinatorial effect we performed isobologram analyses and calculated the combination index, and found that the combination of LT626 and IR was synergistic in both lung and pancreatic cancer cells. SBRT involves the delivery of either a single high dose radiation treatment or a few fractionated radiation treatments. Therefore, we wanted to study the combinatorial effect of LT626 and IR in a fractionated radiation scheme. Our studies showed that cells pretreated with LT626 for 30 minutes followed by irradiation for three consecutive days had significantly lower survival rates than cells treated with LT626 or radiation alone. This shows that just 30 minutes of preincubation with LT626 was enough to sensitize cells to radiation. PARP inhibitor LT626 is similar in structure to BMN673 which is one the most potent PARP trapping drugs [35]. Therefore LT626 may act in a similar fashion as BMN673 by trapping PARP-DNA complex thereby preventing dissociation of PARP from DNA which is an important step necessary for DNA repair [36].

Previously we have shown that PARP inhibition [23] increased the expression of γH2AX in triple negative breast cancer cells. Our current work shows that LT626 and radiation treatment lead to increased γH2AX foci and cell treated in combination with LT626 and radiation had even higher percentage of γH2AX positive cells. Our study showed that radiation treatment led to increased γH2AX foci which peaked after 4–8 h of irradiation and dropped by 24 h, but when cells were treated in combination γH2AX positive cells increased as function of time. This may be attributed again to inefficient DNA repair leading to sustained DNA damage.

53BP1 plays a key role in DNA repair response and checkpoint control. Upon the induction of DNA DSBs, 53BP1 rapidly redistributes from a diffuse nuclear localization to discrete foci that co-localize with phosphorylated histone H2AX and other repair proteins including BRCA1 [37–39]. Bouwman et al. [40] found that a subset of BRCA1-associated human breast cancers have lost 53BP1 protein expression. This loss of 53BP1 in BRCA1-associated cancers may result in resistance to PARP inhibitors and platinum agents. Our current study also looked at the expression of 53BP1 and found that similar to γH2AX pattern 53BP1 nuclear foci increased in a time dependent manner in cells treated with combination therapy.

To validate our *in vitro* observations from our current study, we undertook a series of *in vivo* studies using lung cancer xenograft models. We used the same two cell lines that we had used in our *in vitro* studies, namely H1299 and H460. Similar to a previous study [41] which showed that combination treatment with PARP inhibitor ABT888 (25 mg/kg) and irradiation (2 Gy) for 5 consecutive days delayed tumor growth in H460 xenograft model, our study showed combination treatment with LT626 (10 mg/kg and 20 mg/kg) with radiation (1 Gy) for 5 consecutive days was effective in decreasing the tumor burden and increasing overall survival.

Understanding the molecular mechanism behind drug treatment is critical to predict the clinical efficacy of treatment. Our study showed that LT626 treatment led to increased expression of phosphorylated ATR along with increased expression of phosphorylated Chk1, whereas radiation treatment increased the expression of ATM and Chk2. Interestingly, combination treatment led to increased expression of both ATM and ATR and their kinases. This is similar to other studies [42, 43] which have shown that PARP inhibition in combination with other drugs led to increased expression of ATM. It is possible that ATM is responding to double stranded breaks (DSBs) resulting from irradiation to trigger homologous recombination DNA repair, however inhibition of PARP blocks base excision repair thereby leading to collapse of single stranded breaks to DSBs that can possibly trigger phosphorylation of ATR. Therefore, the levels of DSBs resulting from PARP inhibition combined with DSBs by radiation are detrimental to overall cell survival, this maybe one of the reason why PARP inhibitor may act as a good radiation sensitizer.

In the study presented here, we provide an approach that combines PARP inhibitor with low concentration of radiation to inhibit cell growth in a fractionated radiation scheme. A similar clinical approach can be considered for treatment of lung cancers using PARP inhibitors and SBRT.

## MATERIALS AND METHODS

### Cell lines and reagents

All cell lines were used within 6 months of purchase. H460 (wild type p53), H1299 (p53 null), Miapaca2 (mutations in k-ras and p53) were obtained from the American Type Culture Collection (ATCC). PDA cell line (mutations in k-ras and p53) was a kind gift from Dr. Edgar G. Engleman of Stanford University. H1299 and H460 lung cancer cells were maintained in RPMI1640 with 10% fetal bovine serum (FBS). PDA cells were grown in DMEM with 10% FBS and 1% non-essential amino acids. Miapaca2 cells were maintained in DMEM with 10% FBS and 2.5% horse serum. All cell line media also contained 1% v/v penicillin-streptomycin (100 IU/mL, 100 mg/mL; Thermo Scientific Carlsbad, CA.). All cells were propagated at 37°C in a humidified atmosphere maintained at 5% CO2. PARP inhibitor, LT626 was a gift from BioMarin Pharmaceutical Inc. (San Rafael, CA). Synthesis of LT626 has been described previously by McPherson et al [26]. Solutol HS-15, Dimethyl sulfoxide (DMSO), Methylene blue and MTT was purchased from Sigma Chemicals (St. Louis, MO). N,N-Dimethylacetamide (DMAC) was purchased from EMD Millipore (Billerica, MA).

### Cell viability and colony formation assay

Cell viability was measured by MTT assay. For the MTT assay, cells were plated in 96 well plates and treated with LT626 as indicated for 5 days. After treatment MTT reagent was added to the cells, which was reduced to purple formazan crystals by the mitochondria of living cells. The crystals were solubilized with DMSO and the absorbance was measured at 570 nm by spectrophotometry. All experiments were done at least in triplicate and repeated three independent times. The data were plotted as mean ± SD. Data from a representative experiment is shown in the Figures. For colony formation assay, Miapaca2, PDA, H1299 and H460 cells were irradiated with indicated doses of radiation, using cesium source irradiator. Immediately after treatment, cells were counted and either 100 or 200 cells were plated. After 10–15 days cells were stained with methylene blue and individual colonies were counted. For fractionated radiation study, cells were pretreated with LT626 (0.01–10 μM) for 30 minutes and irradiated (2 Gy) for three consecutive days. On the third days cells were replated for colony formation assay. Student *T*-test was performed to determine statistical significance, groups were compared to their respective controls (no drug control group to LT626 alone and IR alone control group to IR+LT626).

### Combination studies

For combination studies Miapaca2, PDA, H1299 and H460 cells were seeded in 12 well plates (in triplicate). Cells were treated with LT626 or radiation alone or with the combination of LT626 and radiation at the indicated doses. Immediately after radiation 100–200 cells were plated for colony formation assay (as described). The data was plotted using Calcusyn Biosoft software. Combination index (CI) and isobolograms were plotted using the CI equation of Chou-Talalay. A non-constant ratio drug combination design was used to calculate CI values. CI < 1 was synergistic, CI = 1 was additive and CI > 1 was antagonistic. Study was repeated three independent times and representative data are shown.

### γH2AX and 53BP1 staining

PDA, Miapaca2, H1299 and H460 cells were plated overnight in chambered slides (1200 -1500 cells per chamber). Cells were treated with 10 μM LT626 or 2Gy radiation or with combination of both for either 1–24 h for γH2AX or 1–8 h for 53BP1. Controls included primary alone, isotype control and secondary alone. After treatment cells were fixed with 4% paraformaldehyde and stained overnight with primary antibody for γH2AX-Ser139 (1:500 dilution; Cell signaling, Boston, MA) or 53BP1 (1:500 dilution, Bethyl Laboratories, Montgomery, TX). Cells were washed with TBS/ bovine serum albumin and incubated with either Alexa 594 or Alexa 488 (Thermo Scientific) secondary antibody for γH2AX or 53BP1 respectively. Cells were fixed in Prolong gold antifade with DAPI (Thermo Scientific) and cured at room temperature for 24 h before visualization. For quantification of foci at least 100 cells from each treatment group were visually scored. Cells showing more than three foci were counted as positive for γH2AX or 53BP1. The ratio of foci in control versus treated groups was represented as fold change. Images from random fields were taken using Leica DMI6000B with a 40× lens. Student *T*-test was performed to determine statistical significance.

### Western blot analyses

Total cellular protein was isolated using modified radio immunoprecipitation assay buffer. Nuclear extracts were prepared using and following the protocol of NE-PER nuclear and cytoplasmic extraction kit (Thermo Scientific). Protein was separated using either 4–12% Bis-Tris gels or 4–12% Tris-Acetate gels (Thermo Scientific) and transferred to PVDF membranes. Blots were probed with antibodies against ATR (Ser 428), ATM (Ser1981), Chk1 (Ser345), Chk2 (Thr68) (Cell Signaling), actin, cleaved caspase 3 and TBP (Santa Cruz Biotechnology, Santa Cruz, CA). Quantification of blots was carried out using ImageJ software (NIH, https://imagej.nih.gov/ij/) and compared to control.

### H1299 and H460 xenograft studies

All animal care and euthanasia was performed under the approval of the Administrative Panel on Laboratory Animal Care at Stanford University. To establish tumors xenografts, 4 weeks old nude mice (nu/nu, Charles River Laboratories Inc. Wilmington, MA) were subcutaneously inoculated in their right upper shoulder with 1.5 million H1299 or H460 cells. Treatment was started once a tumor reached 150–200 mm^3^ in volume. The mice were randomly assigned into following groups (minimum of 5 mice per group): control, 10 mg/kg LT626 alone, 20 mg/kg LT626 alone, 1Gy IR alone, 10 mg/kg LT626 + IR, 20 mg/kg + IR. For mice receiving LT626 (Gift from BioMarin), a drug formulation utilizing Solutol HS15 and DMAC (Sigma-Aldrich Co. LLC) as previously described was delivered by oral gavage using 1-inch animal feeding needles (Cadence Science Inc., VA, USA). For mice receiving combination treatment, LT626 was administered 30 m prior to radiation. Control and IR group received vehicle control (solutol + DMAC). For radiation treatment mice were anesthetized with a ketamine-xylazine mixture and affixed to a lead shield, exposing only the tumor and underlying tissue to applied radiation. Treatment consisted of a single 1 Gy dose of radiation delivered with a single 225 kV beam using the Kimtron IC 225 irradiator (Kimtron Medical, CT, USA). The dose delivered to the tumors was measured with thermoluminescent dosimeters calibrated in the treatment beam. The mice were treated for 5 consecutive days with this treatment regimen. Following treatment tumors were measured 3 times a week till the end of the study. Survival curve and tumor volume were plotted using PRISM. Statistical analysis using Logrank test for trend was carried out to demonstrate significance (*P* < 0.001) between control and treated groups.

## SUPPLEMENTARY MATERIALS FIGURES AND TABLES



## Abbreviations

SBRT: stereotactic body radiation therapy
PARP: poly (ADP-ribose) polymerase
NSCLC: Non-small cell lung cancer
ATR: Ataxia Telangiectasia And
Rad3: Related Protein
ATM: Ataxia Telangiectasia Mutated
Chk1: Checkpoint Kinase 1
Chk2: Checkpoint Kinase 2
53BP1: Tumor Protein 53-Binding Protein 1
Gy: Grey
IC_50_: half maximal inhibitory concentration
P.O.: Per os
IR: radiation
