# Supplementary material for: Poly (ADP-ribose) polymerase inhibitor, an effective radiosensitizer in lung and pancreatic cancers
Source: Oncotarget. 2017 Feb 17;8(16):26344–55. doi: 10.18632/oncotarget.15464 (PMC5432262; doi:10.18632/oncotarget.15464)
Supplement: Supplementary file 1 [file oncotarget-08-26344-s001.pdf]

Poly (ADP-ribose) polymerase inhibitor, an effective radiosensitizer in lung and pancreatic cancers

Supplementary Materials

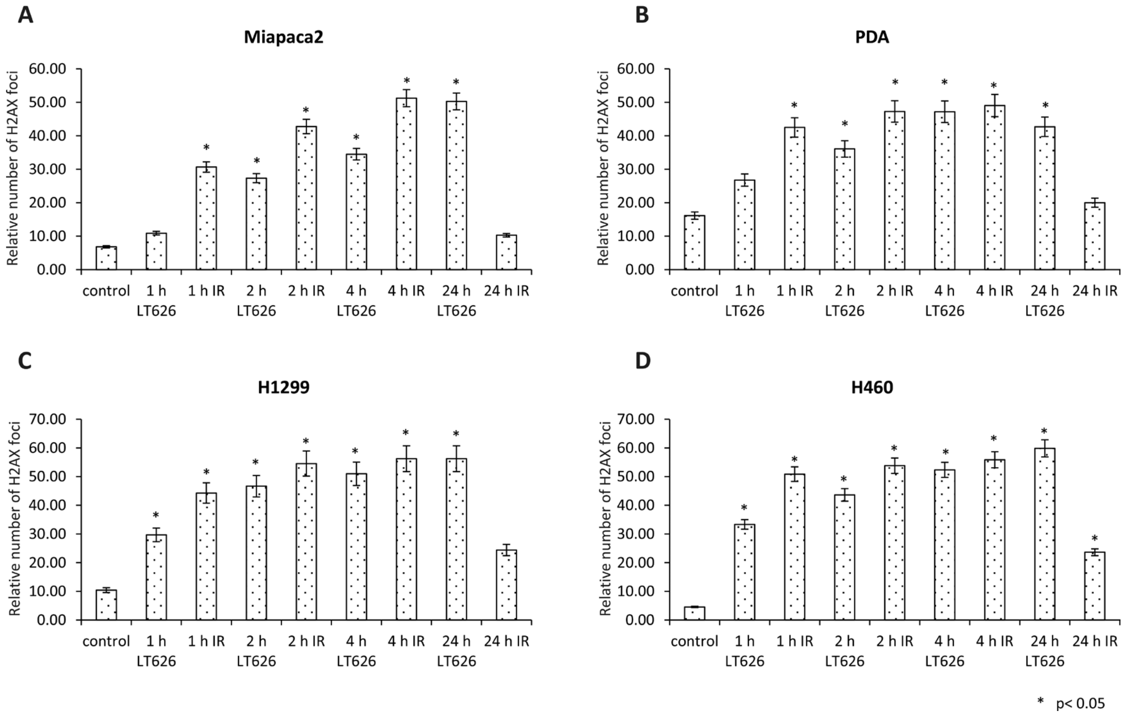

Supplementary Figure 1: Increased expression of γH2AX after LT626 and IR treatment. Pancreatic and lung cancer cell lines were treated with 10 µM LT626 or 2 Gy irradiation for desired time. Following treatment cells were fixed and stained for γH2AX. At least 100 cells were counted and only cells with more than 3 punctate γH2AX foci were considered positive. (**A**) Miapaca2. (**B**) PDA. (**C**) H1299 and (**D**) H460 cells. Experiments were done three independent times in triplicate. Bars represent cell viability ± SD. Statistical analysis to demonstrate significance was performed by using Student's *T*-test.

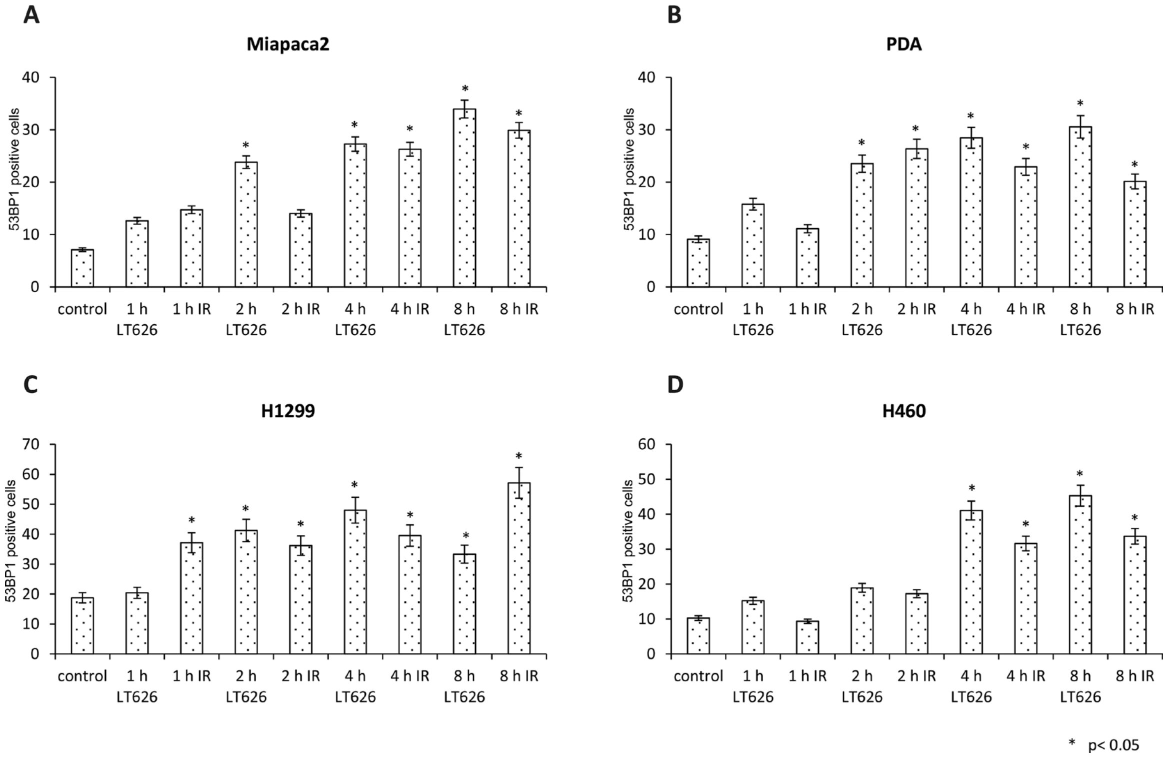

Supplementary Figure 2: Expression of 53BP1 after LT626 and IR treatment. Pancreatic and lung cancer cell lines were treated with 10 µM LT626 or 2 Gy irradiation for desired time. Following treatment cells were fixed and stained for 53BP1. At least 100 cells were counted and only cells positive for punctate nuclear 53BP1 staining were considered positive. (**A**) Miapaca2. (**B**) PDA. (**C**) H1299 and (**D**) H460 cells. Experiments were done three independent times in triplicate. Bars represent cell viability ± SD. Statistical analysis to demonstrate significance was performed by using Student's *T*-test.
